# Analysis of the potential biological mechanisms of Danyu Gukang Pill against osteonecrosis of the femoral head based on network pharmacology

**DOI:** 10.1186/s12906-023-03843-x

**Published:** 2023-01-31

**Authors:** Yongchang Guo, Wenxi Li, Yuju Cao, Xiaoyan Feng, Caihong Shen, Shunguo Gong, Fengzhi Hou, Zhimin Yang, Xifeng Chen, Jingbo Song

**Affiliations:** 1 Department of Orthopedics, Zhengzhou Traditional Chinese Hospital of Orthopaedics, Zhengzhou, 450000 Henan China; 2 Department of Pharmacy, Zhengzhou Traditional Chinese Hospital of Orthopaedics, Zhengzhou, 450000 Henan China

**Keywords:** Danyu Gukang Pill, Osteonecrosis of the femoral head, Network pharmacology, Molecular docking, Active ingredients

## Abstract

**Background:**

Osteonecrosis of the femoral head (ONFH) is still a challenge for orthopedists worldwide and can lead to disability if patients are not treated effectively. Danyu Gukang Pill (DGP), a traditional Chinese medicine (TCM) formulation, is recognized to be effective against ONFH. Nevertheless, its molecular mechanisms remain to be clarified.

**Methods:**

The active ingredients of DGP were collected from the online databases according to oral bioavailability (OB) and drug-likeness (DL). The potential targets of DGP were retrieved from the TCMSP database, while the potential targets of ONFH were obtained from the GeneCards and NCBI databases. The functions and signaling pathways of the common targets of DGP and ONFH were enriched by GO and KEGG analyses. Subsequently, molecular docking and in vitro cell experiments were performed to further validate our findings.

**Results:**

In total, 244 active ingredients of DGP and their corresponding 317 targets were obtained, and 40 ONFH-related targets were predicted. Afterwards, 19 common targets of DGP and ONFH were obtained and used as potential targets for the treatment of ONFH. Finally, combined with network pharmacology analysis, molecular docking and in vitro cell experiments, our study first demonstrated that the treatment effect of DGP on ONFH might be closely related to the two targets, HIF1A (HIF-1α) and VEGFA, and the HIF-1 signaling pathway.

**Conclusions:**

This study is the first to investigate the molecular mechanisms of DGP in the treatment of ONFH based on network pharmacology. The results showed that DGP might up-regulate the expression of HIF-1α and VEGFA by participating in the HIF-1 signaling pathway, thus playing an anti-ONFH role.

**Supplementary Information:**

The online version contains supplementary material available at 10.1186/s12906-023-03843-x.

## Introduction

Osteonecrosis of the femoral head (ONFH) is a common progressive disease characterized by reduced blood supply to the femoral head, bone metabolic disorders, necrosis of the subchondral bone, impaired microcirculation, interrupted bone remodeling processes, and eventually resulting in femoral head collapse [1, 2]. In China, there are approximately 8.12 million cases of ONFH in the cohort over the age of 15, and about 11.76 cases of ONFH per 100,000 people in the general rural population, while 9.57 ONFH patients per 100,000 people in the urban population. It has been reported that without effective treatment, about 80% of ONFH patients will suffer femoral head collapse within 1–4 years, resulting in necrosis or disability, and some patients will even have to undergo total hip arthroplasty (THA) [3, 4]. Therefore, ONFH has always been regarded as a major problem in the field of orthopedics. Currently, the commonly used drugs for the treatment of ONFH are bisphosphonates, statins and anticoagulants [5], which can partially restore the function of bone cells through different pharmacological mechanisms, but their obvious side effects, such as gastrointestinal irritation, nephrotoxicity or mandibular joint necrosis, should not be ignored. Complementary and alternative medicine (CAM), such as traditional Chinese medicine (TCM), has been used by a large number of individuals to treat ONFH [6–9].

Danyu Gukang Pill (DGP) has the effects of promoting blood circulation, removing blood stasis, dredging meridians, relieving pain, tonifying kidney, and strengthening bones, and is mainly used to treat ONFH. DGP is composed of 19 traditional Chinese medicines, including *Panax notoginseng* (Burk.) F. H. Chen (San Qi), *Spatholobus suberectus* Dunn (Ji Xue Teng), *Achyranthes bidentata* Bl. (Niu Xi), *Dipsacus asper* Wall (Xu Duan), *Drynaria fortunei* (Kunze) J. Sm. (Gu Sui Bu), *Lycium barbarum* L. (Gou Qi Zi), *Salvia miltiorrhiza* Bge*.* (Dan Shen), *Angelica sinensis* (Oliv.) Diels (Dang Gui), *Ligusticum chuanxiong* Hort. (Chuan Xiong), *Acanthopanax gracilistylus* W. W. Smith (Wu Jia Pi), *Rehmannia glutinosa* Libosch. (Shu Di Huang), *Codonopsis tangshen* Oliv. (Dang Shen), *Atractylodes macrocephala* Koidz. (Bai Zhu), *Boswellia carterii* Birdw. (Ru Xiang), *Commiphora myrrha* (Nees) Engl. (Mo Yao), *Corydalis yanhusuo* W. T. Wang (Yan Hu Suo), *Rheum officinale* Baill. (Da Huang), *Curcuma wenyujin* Y. H. Chen (Yu Jin), and *Aucklandia lappa* Decne. (Mu Xiang). Previous clinical trials have shown that DGP is a good medicine for ONFH, and the X-ray imaging and efficacy, such as the improvement in analgesia and claudication, in the DGP group are superior to those in the control group [10]. In addition, one relevant study also showed that the interventional administration of DGP can effectively reduce the incidence of ONFH after the operation on femoral neck fracture, as well as relieve the degree of hip pain, and improve hip function within one year after the operation [11]. However, its specific mechanisms remain to be further studied.

Network pharmacology is a new approach that combines systems biology, pharmacokinetics and pharmacodynamics, and can systematically reveal the biological mechanism of action of drugs in complex diseases at the molecular level [12–14]. This method focuses on the synergistic effects of multi-components and multi-targets, which accords with the overall concept of the TCM treatment of diseases. At present, network pharmacology is increasingly used to study the therapeutic mechanisms of TCM in various diseases.

**Fig. 1:**
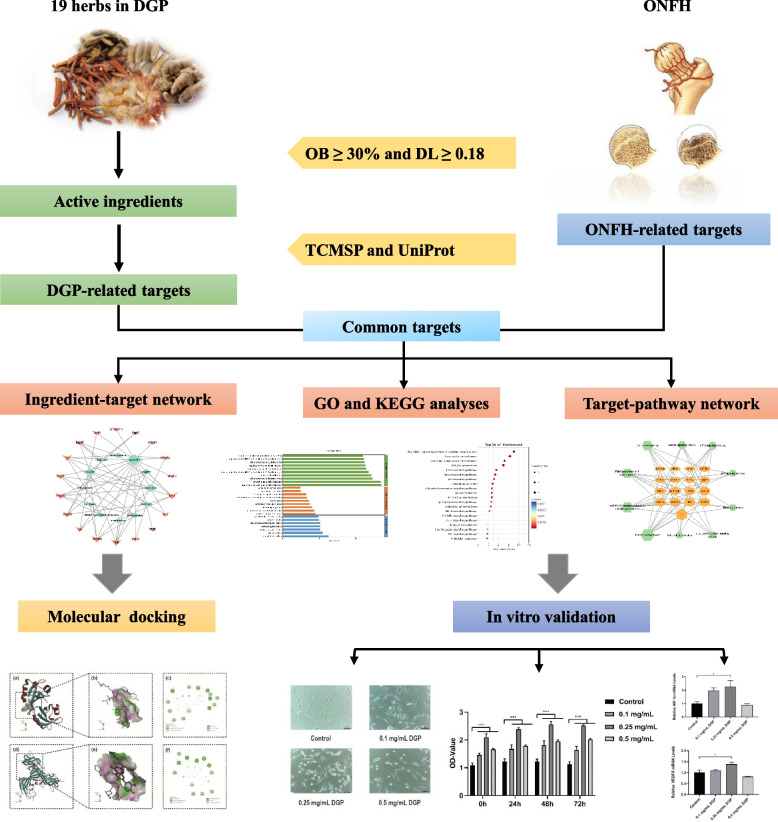
Flow chart of network pharmacology analysis of DGP against ONFH

In the current study, we used the approaches of network pharmacology and molecular docking to investigate the potential molecular mechanisms of DGP against ONFH. Additionally, human bone marrow mesenchymal stem cells (BMSCs) play a key role in maintaining the structural and functional integrity of the femoral head to prevent ONFH due to their proliferative capacity and osteogenic differentiation. Therefore, in this study, BMSCs were used for in vitro experiments to verify the potential mechanism of DGP against ONFH. The detailed technical strategy of this study is shown in Fig. 1.

## Materials and methods

### Acquisition of DGP-related active ingredients

All ingredients of 19 Chinese herbs in DGP were obtained from the Traditional Chinese Medicine Systems Pharmacology Database and Analysis Platform (TCMSP, https://old.tcmsp-e.com/index.php) and Bioinformatics Analysis Tool for Molecular mechANism of Traditional Chinese Medicine (BATMAN-TCM, http://bionet.ncpsb.org/batman-tcm/index.php) databases based on the screening criteria of oral bioavailability (OB) ≥ 30% and drug-likeness (DL) ≥ 0.18.

### Prediction of DGP-related targets

We input all active ingredients into the TCMSP database to predict the potential targets of DGP and set the species as “Homo sapiens”. Meanwhile, the UniProt database (https://www.uniprot.org/) was used to standardize the targets of each active ingredient.

### Collection of ONFH-related targets

The targets associated with ONFH were collected from the NCBI (https://www.ncbi.nlm.nih.gov/gene/) and GeneCards (https://www.genecards.org) databases. We searched for targets from these two databases using the keyword “osteonecrosis of the femoral head”, and then obtained the common targets in the NCBI and GeneCards databases as ONFH-related targets.

### Protein–protein interaction (PPI)

The online analysis tool Venny 2.1 was used to obtain potential targets of DGP for the treatment of ONFH. The DGP-ONFH common target list was entered into the STRING database (https://cn.string-db.org/) for the construction of PPI network, and then this network was visualized using Cytoscape software.

### GO and KEGG analyses

Gene Ontology (GO) functional annotation and Kyoto Encyclopedia of Genes and Genomes (KEGG) signaling pathway enrichment analysis were performed using the online analysis tools of the bioinformatics platform (http://www.bioinformatics.com.cn/).

### Construction of networks

The ingredient-target network and target-pathway network were constructed by Cytoscape software. In these networks, nodes represent ingredients, targets, or signaling pathways, and edges represent the interactions between them.

### Molecular docking

The 3D molecular structure of active ingredients was downloaded from the PubChem database (https://pubchem.ncbi.nlm.nih.gov/). The crystal structure of target proteins was obtained from the RCSB Protein Data Bank (PDB, http://www.rcsb.org/). Subsequently, molecular docking of ligands with receptors was conducted by AutoDock Vina software. At last, the results of molecular docking were analyzed using Discovery Studio 2016 Client and PyMOL.

## Experimental validation

### Preparation of DGP aqueous solution

DGP (0.2 g) was dissolved in 40 mL double-distilled water to prepare a 5 mg/mL stock solution, which was then diluted to the required concentration for reserve.

### Cell culture

BMSCs were bought from Procell Life Science&Technology Co., Ltd (Cat No.: CP-H166, Wuhan, China), and were cultured in the Minimum Essential Medium (MEM)-alpha medium with 10% Foetal Bovine Serum (FBS), 100 U/mL penicillin, and 100 mg/mL streptomycin. Afterwards, cells were kept at 37 °C with 5% CO_2_.

### Cell viability assay

Cell viability was determined by Cell Counting Kit-8 (CCK-8) assay. The cell density in the control and DGP groups was adjusted to 5 × 10^3^, and then cells were inoculated into 96-well plates. After cells were fully attached, they were then treated with different concentrations of DGP aqueous solution (0 mg/mL, 0.1 mg/mL, 0.25 mg/mL, and 0.5 mg/mL) for 12, 24, 36, and 48 h. Afterwards, CCK-8 (5 mg/mL) was added to each well and the cells were incubated at 37 °C for 4 h. We then discarded the supernatant and added dimethyl sulfoxide (DMSO). Optical density (OD) at 570 nm was measured using a microplate reader. The experiments were repeated three times independently.

### Quantitative real-time Polymerase Chain Reaction (qRT-PCR)

Total RNA was extracted from cells using TRIzol reagent (Invitrogen, Carlsbad, CA, USA) according to the manufacturer’s instructions. The One Step PrimeScript miRNA cDNA Synthesis Kit (TaKaRa Biotechnology, Dalian, China) was used to synthesize the cDNA of genes. The qRT-PCR was performed using the ABI 7500 Sequence Detection System (Applied Biosystems, Foster City, CA, USA). The mRNA expression of HIF-1α and VEGFA was normalized using GAPDH as an internal control. The primer sequences for PCR were: HIF-1α: F—5’-GCCTCTGTGATGAGGCTTACC-3’, R—5’-CAGTGCAATACCTTCCATGTTGC-3’; VEGFA: F—5’-ACTGCCATCCAATCGAGACC-3’, R—5’-TTGATCCGCATAATCTGCATGGT-3’; and GAPDH: F—5’-GAGTCAACGGATTTGGTCGT-3’, R—5’-GACAAGCTTCCCGTTCTCAG-3’. The results were calculated using the 2^−ΔΔCt^ equation.

### Statistical analysis

Data are presented as mean ± standard deviation (SD). SPSS version 20.0 was used to analyze statistical differences. The significance level was *p* < 0.05 for all statistical tests.

## Results

### Identification of bioactive ingredients and targets of DGP

In total, 2,350 ingredients in DGP were retrieved from the TCMSP and BATMAN-TCM databases, while 371 were selected based on the screening criteria (OB ≥ 30% and DL ≥ 0.18) (Table 1). After excluding ingredients without corresponding targets and duplicates, 244 ingredients and their corresponding 317 targets were finally collected from the TCMSP database for next analyses.

**Table 1:** Number of ingredients in DGP with OB ≥ 30% and DL ≥ 0.18

Number	Chinese name	Latin binomial nomenclature name	Total	OB ≥ 30% and DL ≥ 0.18
1	San Qi	*Panax notoginseng* (Burk.) F. H. Chen	119	8
2	Ji Xue Teng	*Spatholobus suberectus* Dunn	68	24
3	Niu Xi	*Achyranthes bidentata* Bl	176	20
4	Xu Duan	*Dipsacus asper* Wall	31	8
5	Gu Sui Bu	*Drynaria fortunei* (Kunze) J. Sm	71	18
6	Gou Qi Zi	*Lycium barbarum* L	188	45
7	Dan Shen	*Salvia miltiorrhiza Bge*	202	65
8	Dang Gui	*Angelica sinensis* (Oliv.) Diels	125	2
9	Chuan Xiong	*Ligusticum chuanxiong* Hort	189	7
10	Wu Jia Pi	*Acanthopanax gracilistylus* W. W. Smith	16	5
11	Shu Di Huang	*Rehmannia glutinosa* Libosch	76	2
12	Dang Shen	*Codonopsis tangshen* Oliv	134	21
13	Bai Zhu	*Atractylodes macrocephala* Koidz	55	7
14	Ru Xiang	*Boswellia carterii* Birdw	127	8
15	Mo Yao	*Commiphora myrrha* (Nees) Engl	276	45
16	Yan Hu Suo	*Corydalis yanhusuo* W. T. Wang	77	49
17	Da Huang	*Rheum officinale* Baill	92	16
18	Yu Jin	*Curcuma wenyujin* Y. H. Chen	222	15
19	Mu Xiang	*Aucklandia lappa* Decne	106	6

### Identification of ONFH-related targets

A total of 97 and 442 ONFH-related targets in Homo sapiens were collected from the NCBI and GeneCards databases, respectively. After filtering targets in the GeneCards database by twice the median “relevance score”, 105 ONFH-related targets were screened. Finally, 40 ONFH-related targets were obtained by intersecting the targets from the NCBI and GeneCards databases.

### Acquisition of DGP-ONFH common targets and construction of PPI network

**Fig. 2:**
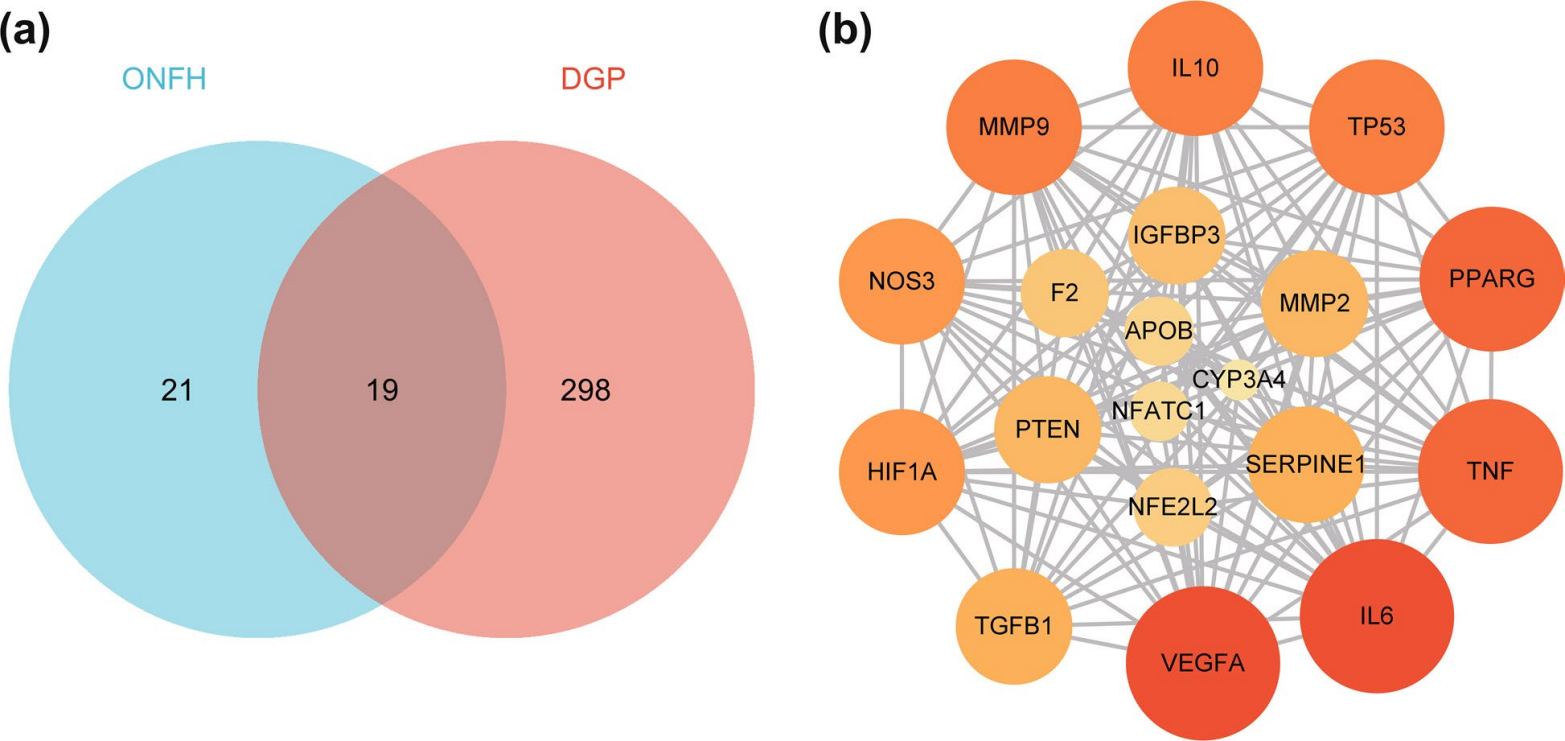
**a** Venn diagram of DGP-related targets and ONFH-related targets. **b** PPI network

As shown in Fig. 2a, all DGP- and ONFH-related targets are listed as two separate sets. A Venn diagram represents two sets and their relationship. As a result, 19 common targets of DGP and ONFH were obtained as candidate therapeutic targets of DGP for the treatment of ONFH. Next, the PPI network was constructed through the STRING database and visualized using Cytoscape software. There are 19 nodes in the PPI network, and the nodes with the highest degree value are VEGFA, IL6 and TNF. The information about 19 targets and the degree of interaction between them are shown in Table 2 and Fig. 2b.

**Table 2:** DGP-ONFH common targets

Number	Protein Name	Gene Symbol	Gene ID	Degree
1	Vascular endothelial growth factor A	VEGFA	ENSP00000478570	18
2	Interleukin-6	IL6	ENSP00000385675	18
3	Tumor necrosis factor	TNF	ENSP00000398698	17
4	Peroxisome proliferator activated receptor gamma	PPARG	ENSP00000287820	17
5	Interleukin-10	IL10	ENSP00000412237	16
6	Cellular tumor antigen p53	TP53	ENSP00000269305	16
7	Matrix metalloproteinase-9	MMP9	ENSP00000361405	16
8	Nitric oxide synthase, endothelial	NOS3	ENSP00000297494	15
9	Hypoxia-inducible factor 1-alpha	HIF1A	ENSP00000437955	15
10	Plasminogen activator inhibitor 1	SERPINE1	ENSP00000223095	14
11	Transforming growth factor beta-1	TGFB1	ENSP00000221930	14
12	72 kDa type IV collagenase	MMP2	ENSP00000219070	13
13	Phosphatase and tensin homolog	PTEN	ENSP00000361021	13
14	Insulin-like growth factor-binding protein 3	IGFBP3	ENSP00000370473	12
15	Transcription factor E2F2	F2	ENSP00000308541	11
16	Nuclear factor erythroid 2-related factor 2	NFE2L2	ENSP00000380252	10
17	Apolipoprotein B-100	APOB	ENSP00000233242	9
18	Nuclear factor of activated T-cells, cytoplasmic 1	NFATC1	ENSP00000389377	8
19	Cytochrome P450 3A4	CYP3A4	ENSP00000337915	6

### Construction and analysis of the ingredient-target network

**Fig. 3:**
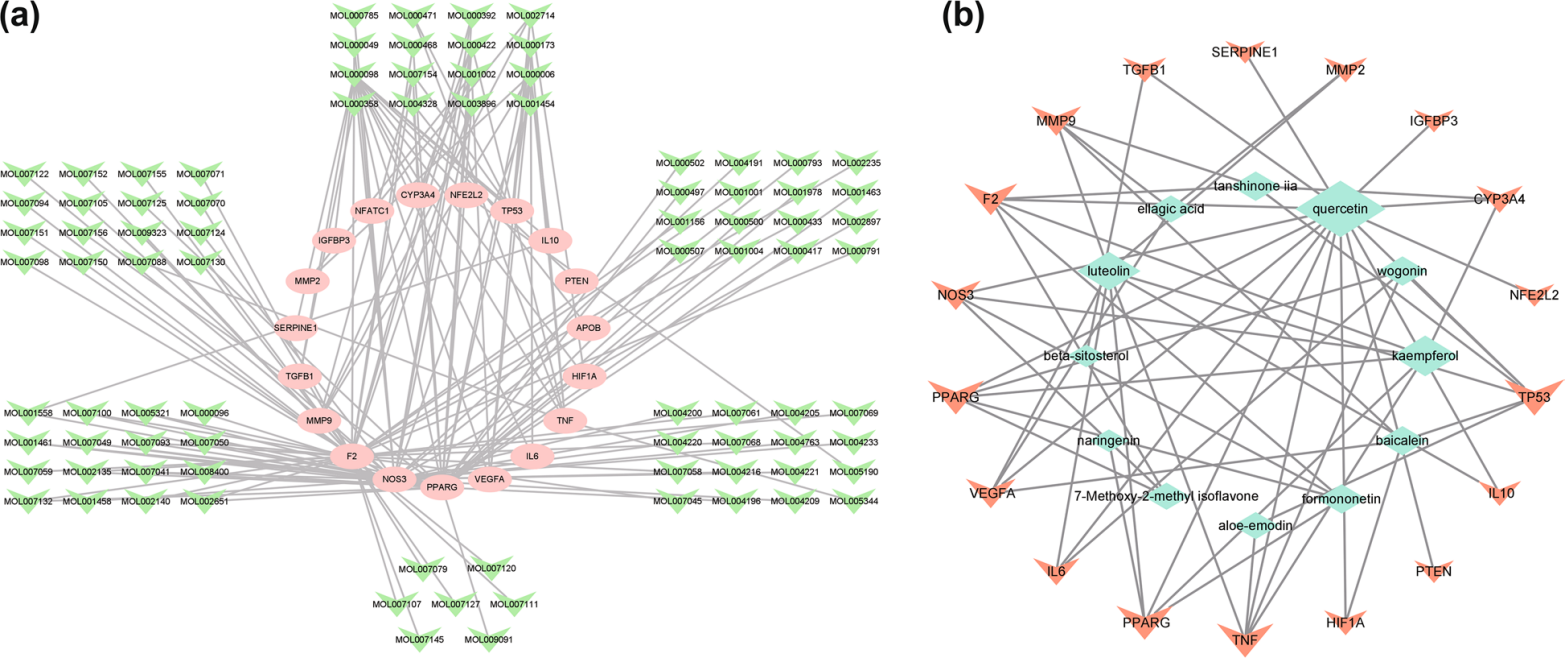
**a** The ingredient-target network of DGP against ONFH. **b** The ingredient-target network of DGP against ONFH (degree value > twice the median “degree value”)

An ingredient-target network (Fig. 3a) was constructed by Cytoscape, which included 106 nodes (87 for bioactive ingredients and 19 for targets). At the same time, we reconstructed the ingredient-target network (Fig. 3b) with twice the median “degree value” as the filtering condition. As a result, we found that several components had higher degree values, mainly including quercetin (MOL000098), luteolin (MOL000006), and kaempferol (MOL000422). We speculated that these components of DGP, especially quercetin, might play a very important role in the treatment of ONFH.

### Results of GO functional annotation and KEGG signaling pathway enrichment

**Fig. 4:**
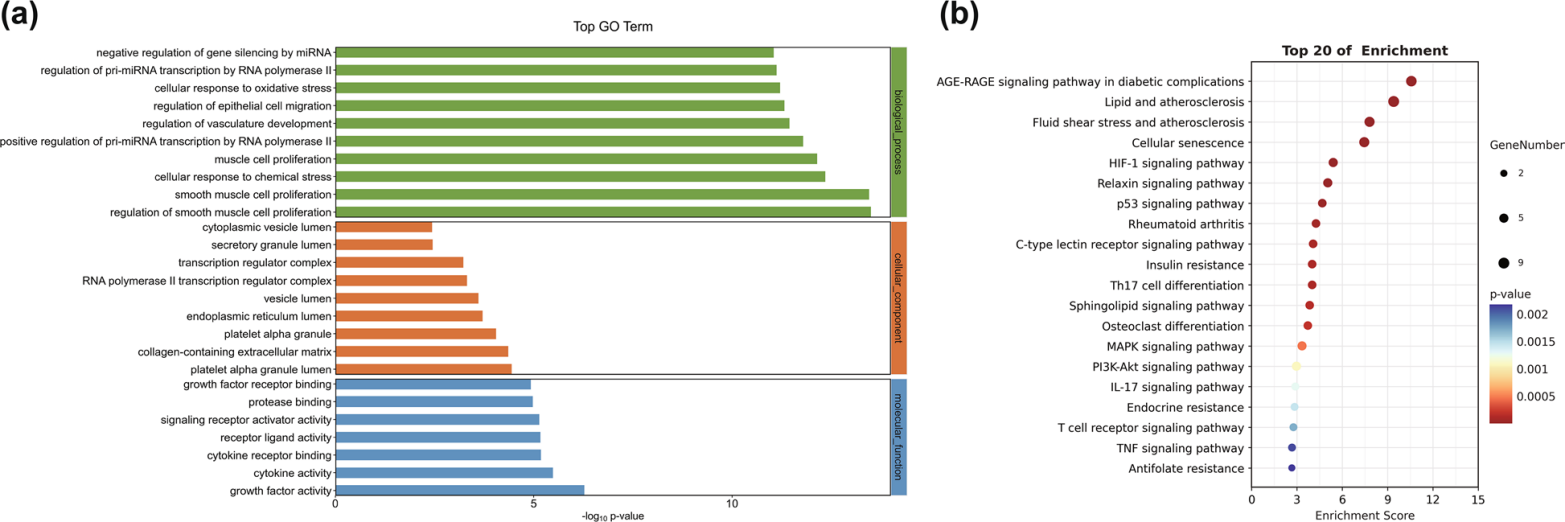
**a** GO functional annotation and **b** KEGG signaling pathway enrichment analysis of DGP-ONFH common targets

The common targets were enriched in 1,839 GO terms, including 1,701 for biological process (BP) terms, 37 for cellular component (CC) terms, and 101 for molecular function (MF) terms (*p* < 0.05). The top 10 GO terms for each category are shown in the bar chart (Fig. 4a). Specifically, the top 3 BP terms were regulation of smooth muscle cell proliferation, smooth muscle cell proliferation, and cellular response to chemical stress. The top 3 CC terms included platelet alpha granule lumen, collagen-containing extracellular matrix, and platelet alpha granule. The top 3 MF terms were growth factor activity, cytokine activity and cytokine receptor binding. Meanwhile, KEGG enrichment analysis indicated that the 19 common targets were significantly enriched in 91 pathways (*p* < 0.05). According to relevant literature, 36 signaling pathways related to OFFH were finally obtained. The first 20 important pathways are shown in Fig. 4b and Supplementary file 1, among which the HIF-1 signaling pathway that was highly ranked and closely related to ONFH was selected for further study.

### Analysis of the target-pathway network

**Fig. 5:**
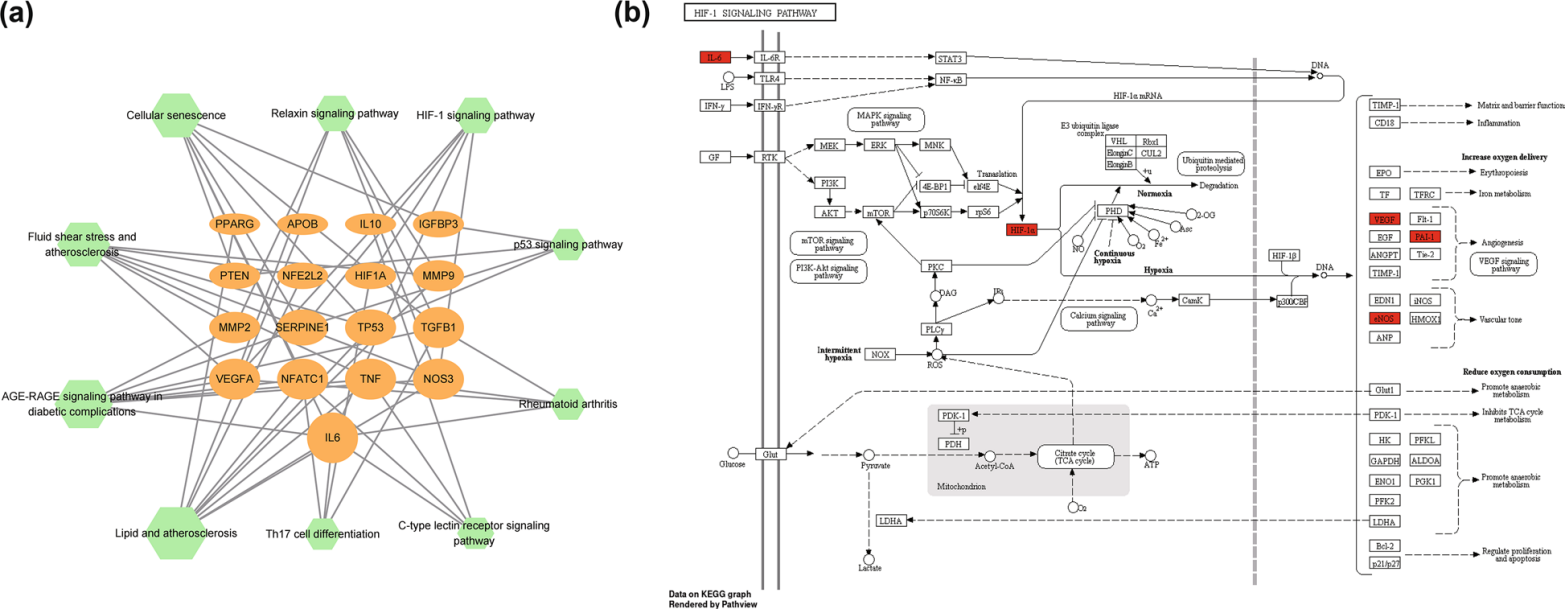
**a** The target-signaling pathway network. The green hexagon nodes represent pathways and the orange ellipse nodes represent targets. **b** The HIF-1 signaling pathway

In order to further clarify the molecular mechanisms of DGP in the treatment of ONFH, we constructed a target-pathway network. As shown in Fig. 5a, this network includes 27 nodes (17 for targets and 10 for signaling pathways). Combined with the PPI network (VEGFA had the best degree value), KEGG signaling pathway enrichment analysis (The HIF-1 signaling pathway ranked higher and was closely related to ONFH) as well as the target-pathway network (Both VEGFA and HIF-1α were enriched in the HIF-1 signaling pathway), we speculated that DGP might regulate the expression of VEGFA and HIF-1α by participating in the HIF-1 signaling pathway, thereby playing a role in the treatment of ONFH. The image of HIF-1 signaling pathway was obtained from KEGG database [15–17] (https://www.kegg.jp/kegg/kegg1.html), and its details are shown in Fig. 5b.

### Results of molecular docking

**Fig. 6:**
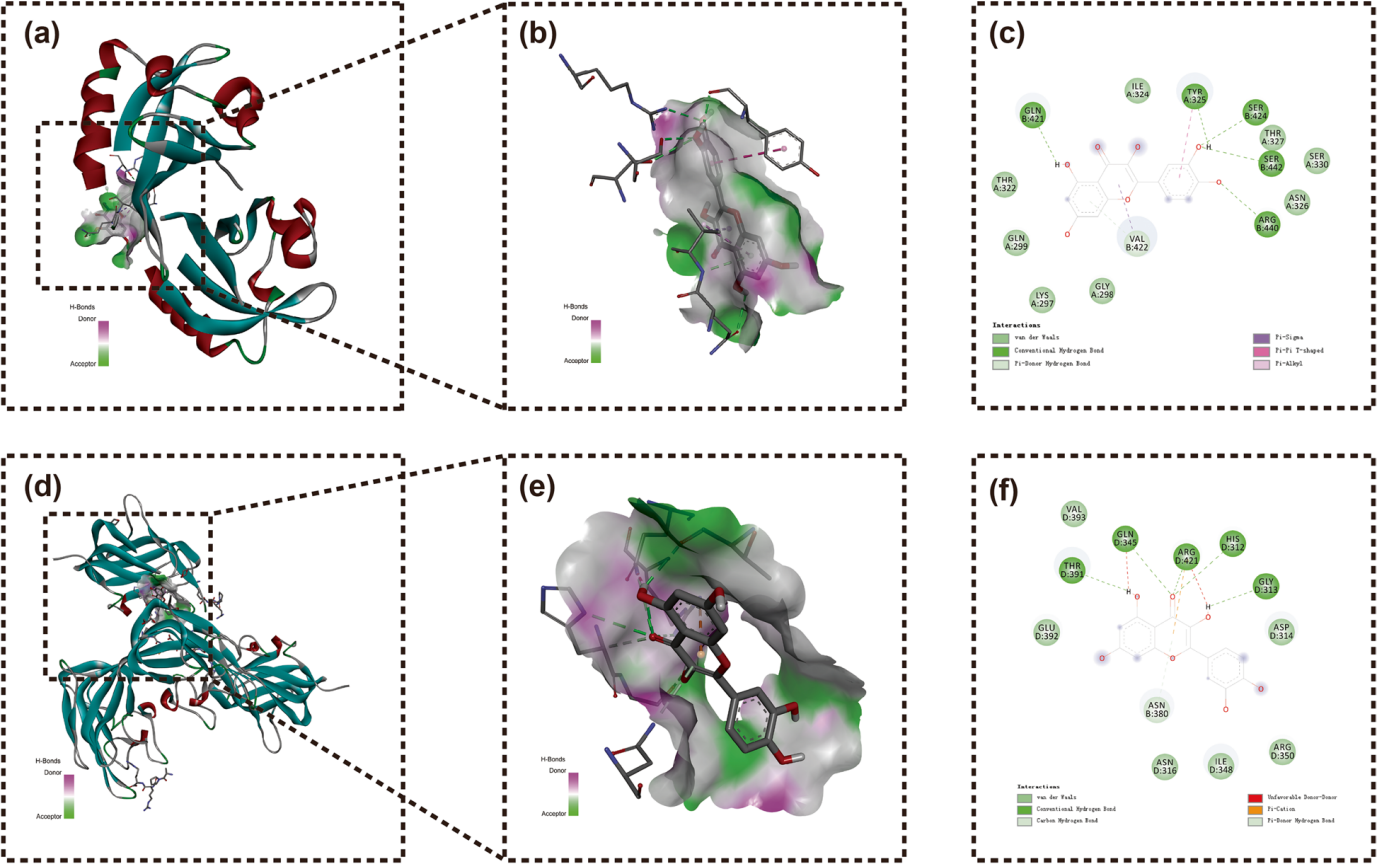
Molecular docking diagram. Docking of quercetin with (**a**-**c**) HIF-1α and (**d**-**f**) VEGFA

To investigate the reliability of molecule-target interactions and accurate binding patterns, we chose quercetin as a crucial molecule and HIF-1α and VEGFA as key targets according to the above results. The lower the binding energy, the more stable the ligand-receptor binding conformation. Binding energy below or equal to -5 kcal/mol represented a good binding ability between molecules and proteins. Our results showed that quercetin had strong interactions with HIF-1α and VEGFA with binding energies of -7.2 kcal/mol and -7.4 kcal/mol, respectively (Fig. 6). This further suggested that the binding activity between the core active ingredient and core target proteins was stable.

## Experimental validation

### DGP could improve the viability of BMSCs

**Fig. 7:**
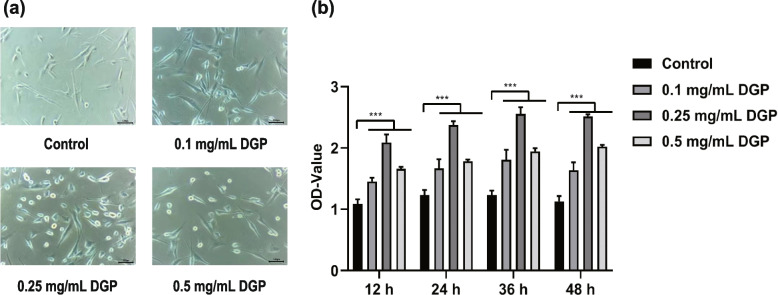
Effects of DGP on BMSCs viability. **a** The representative images of BMSCs morphology under inverted microscope after 24 h of DGP treatment. **b** BMSCs were treated with the specified concentrations of DGP for 12, 24, 36, and 48 h, and cell viability was tested using CCK-8 assay. The results were presented as the mean ± SD of three independent replicates. ^***^p < 0.001

The viability of BMSCs increased significantly after 24 h of DGP treatment (Fig. 7a). The results of CCK-8 assay indicated that treatment with 0.1 mg/mL, 0.25 mg/mL, and 0.5 mg/mL DGP for 12, 24, 36, and 48 h could promote the proliferation of BMSCs compared with the control group. Notably, treatment with 0.25 mg/mL DGP obtained surprisingly the best efficacy (Fig. 7b).

### DGP could increase the mRNA expression of HIF-1α and VEGFA

**Fig. 8:**
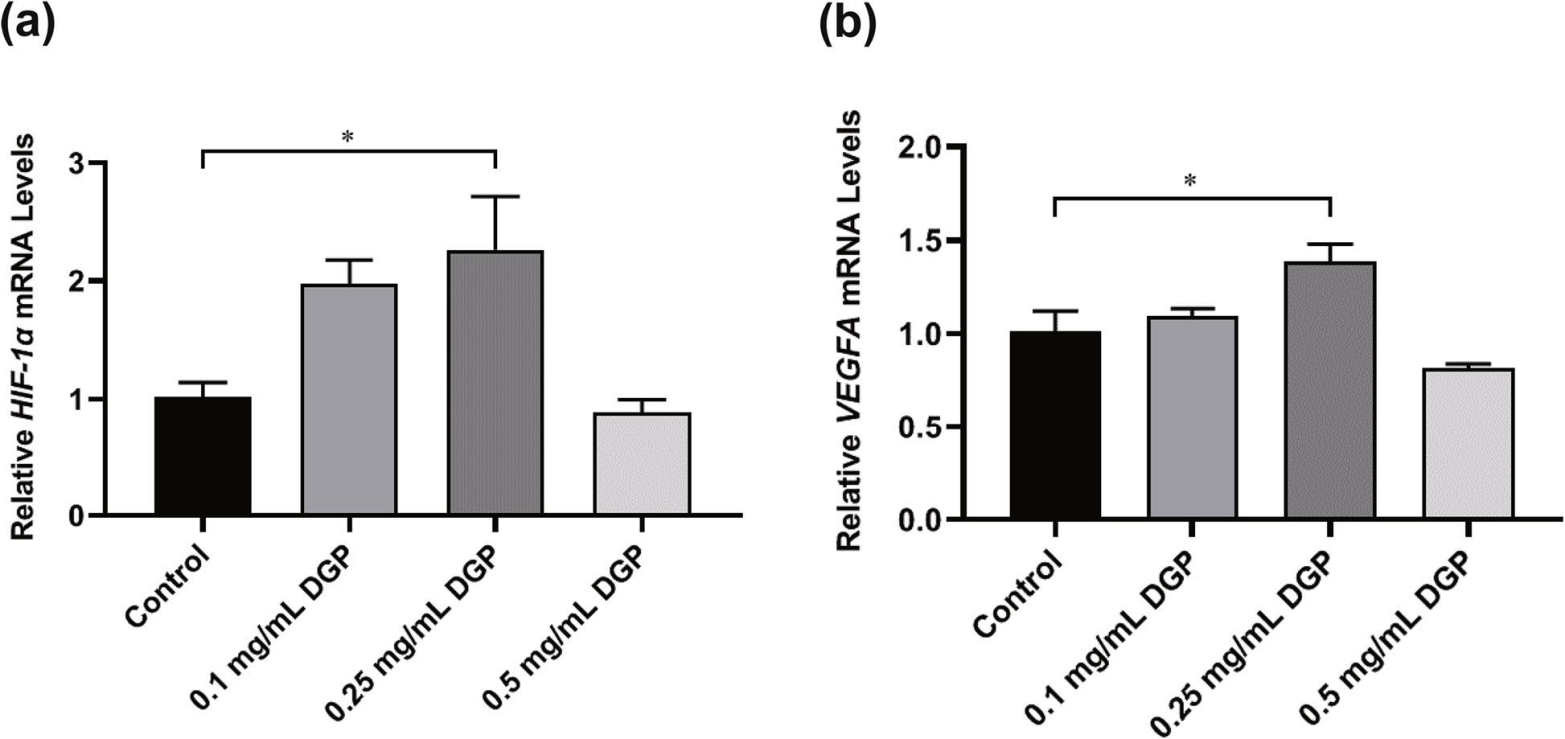
The mRNA expression of (**a**) HIF-1α and (**b**) VEGFA after 24 h of treatment with different concentrations of DGP. The results were presented as the mean ± SD of three independent replicates. ^*^p < 0.05

The qRT-PCR assay demonstrated that treatment with 0.1 mg/mL and 0.25 mg/mL DGP could increase the mRNA expression of HIF-1α (Fig. 8a) and VEGFA (Fig. 8b) to varying degrees. In particular, 0.25 mg/mL DGP could significantly increase the mRNA expression of HIF-1α and VEGFA, while 0.5 mg/mL DGP had the opposite effect.

## Discussion

ONFH is a refractory disease characterized by damaged subchondral microcirculation, skeletal necrosis, and microfracture accumulation without continuous remodeling [18]. The pathological mechanisms of ONFH are very complicated, which are associated with multiple targets and pathways during its development [19]. TCM is commonly composed of a variety of ingredients, and has a wide range of pharmacological activities and various targets and pathways [20]. Accumulating evidence indicates that TCM may be beneficial to the treatment of ONFH [6, 21, 22]. Nevertheless, the characteristics of TCM may make it difficult to further study the underlying mechanisms of drugs in treating diseases. Network pharmacology is an organic combination of systems biology and omics, which can provide a direction for the research on the mechanisms of complex TCM [23]. In this research, we used this method to elucidate the pharmacological mechanisms of DGP in the treatment of ONFH.

It is believed that ingredients lacking appropriate pharmacokinetic properties cannot arrive to the target organ and then transmit biological activity. Thus, we screened active ingredients of DGP based on OB ≥ 30% and DL ≥ 0.18 [24]. Importantly, it has been recognized that ingredients with a high degree value may have the therapeutic effect on ONFH. In our research, quercetin was the most important ingredient with a high degree-value. Previous research has shown that quercetin is a kind of natural flavonoid, and has antibacterial, anti-inflammatory, antioxidative, immunomodulatory, anticancer, anti- atherosclerotic, lipid-modulating and bone-conserving properties. Additionally, quercetin has been reported to inhibit osteoclastogenesis, osteoblast apoptosis, and inflammatory responses, and promote osteogenesis, angiogenesis, adipocyte apoptosis, and osteoclast apoptosis [25]. Just as the study by Pang XG and Zhou Y has demonstrated that quercetin promotes BMSCs proliferation and osteogenic differentiation [26, 27], suggesting that quercetin may play an important role in the treatment of ONFH.

As predicted, DGP might exert its pharmacological effects on the treatment of ONFH by participating in the HIF-1 signaling pathway and targeting HIF-1α and VEGFA. Studies have shown that the functional HIF-1 is composed of two subunits, HIF-1α and HIF-1β, of which HIF-1α is a pivotal regulator of the adaptive response of cells to hypoxia [28, 29]. It has been reported that overexpression of HIF-1α can promote the differentiation of BMSCs into osteoblasts after osteogenic induction and enhance the secretion of VEGF in BMSCs [30, 31]. Furthermore, studies have suggested that BMSCs transplanted into the necrotic femoral head under hypoxia can survive, proliferate and differentiate into osteoblasts to promote osteogenesis. Meanwhile, in vitro exposure of BMSCs to hypoxia can lead to up-regulation of VEGF, thus promoting angiogenesis [32, 33]. Indeed, HIF-1α transfection can enhance the ability of BMSCs to promote osteogenesis and angiogenesis in vitro and protect against ONFH [34, 35]. Therefore, we considered that HIF-1α and VEGFA played an indispensable role in the treatment of ONFH. Remarkably, our in vitro experiments showed that DGP could significantly increase the viability of BMSCs and the mRNA expression of HIF-1α and VEGFA in the HIF-1 signaling pathway, which further verified our hypothesis.

In conclusion, on the basis of the network pharmacological analysis, we further verified our results by molecular docking and in vitro cell experiments. Molecular docking results showed that the binding energies between the effective chemical active ingredient of DGP (quercetin) and key target proteins (HIF-1α and VEGFA) were less than -5 kcal/mol, suggesting that stable binding of quercetin to HIF-1α and VEGFA might be critical in the treatment of ONFH. In vitro cell experiments revealed that DGP could up-regulate the expression of HIF-1α and VEGFA in the HIF-1 signaling pathway, indicating that DGP might treat ONFH by regulating the expression of HIF-1α and VEGFA in the HIF-1 signaling pathway. However, the present study still has some limitations, and the in-depth study of the underlying molecular mechanisms needs to be further explored and verified by in vivo experiments.

## Conclusion

In this study, the potential targets and pathways related to DGP in the treatment of ONFH were systematically analyzed, and the results showed that DGP might regulate the expression of HIF-1α and VEGFA in the HIF-1 signaling pathway, thus playing a crucial role in the treatment of ONFH.

## Supplementary Information

**Additional file 1.**

## Data Availability

The data generated in this study are available from the corresponding author upon request.

## Abbreviations

DGP: Danyu Gukang Pill
ONFH: Osteonecrosis of the femoral head
OB: Oral bioavailability
DL: Drug-likeness
GO: Gene Ontology
KEGG: Kyoto Encyclopedia of Genes and Genomes
BMSCs: Bone marrow mesenchymal stem cells
HIF-1α: Hypoxia-inducible factor-1 alpha
VEGFA: Vascular endothelial growth factor A

## References

[1] Wu H, Cheng K, Tong L, Wang Y, Yang W, Sun Z (2022). Knowledge structure and emerging trends on osteonecrosis of the femoral head: a bibliometric and visualized study. J Orthop Surg Res.

[2] Mont MA, Salem HS, Piuzzi NS, Goodman SB, Jones LC (2020). Nontraumatic osteonecrosis of the femoral head: Where Do We Stand Today?: a 5-year update. J Bone Joint Surg Am.

[3] Papakostidis  C, Tosounidis  TH, Jones  E, Giannoudis  PV (2016). The role of "cell therapy" in osteonecrosis of the femoral head. a systematic review of the literature and meta-analysis of 7 studies. Acta Orthop.

[4] Gao YS, Ai ZS, Zhu ZH, Yu XW, Zhang CQ (2013). Injury-to-surgery interval does not affect postfracture osteonecrosis of the femoral head in young adults: a systematic review. European journal of orthopaedic surgery & traumatology : orthopedie traumatologie.

[5] Larson E, Jones LC, Goodman SB, Koo KH, Cui Q (2018). Early-stage osteonecrosis of the femoral head: where are we and where are we going in year 2018?. Int Orthop.

[6] Yeh YA, Chiang JH, Wu MY, Tsai CH, Hsu HC, Hsu HC (2019). Association of traditional chinese medicine therapy with risk of total hip replacement in patients with nontraumatic osteonecrosis of the femoral head: a population-based cohort study. Evid Based Complement Alternat Med.

[7] Zhang XY, Li HN, Chen F, Chen YP, Chai Y, Liao JZ (2021). Icariin regulates miR-23a-3p-mediated osteogenic differentiation of BMSCs via BMP-2/Smad5/Runx2 and WNT/β-catenin pathways in osteonecrosis of the femoral head. Saudi Pharm J.

[8] Fu F, Huang Z, Ye H, Tan B, Wang R, Chen W (2020). Mechanisms and molecular targets of the Tao-Hong-Si-Wu-Tang formula for treatment of osteonecrosis of femoral head: a network pharmacology study. Evid Based Complement Alternat Med.

[9] Fang B, Li Y, Chen C, Wei Q, Zheng J, Liu Y (2019). Huo Xue Tong Luo capsule ameliorates osteonecrosis of femoral head through inhibiting lncRNA-Miat. J Ethnopharmacol.

[10] Jirong G, Baojun W, Heming W (2000). Summary of Phase II clinical trial of Danyu Gukang Pill for treatment of 90 cases of avascular necrosis of femoral head. Journal of Fujian College of Traditional Chinese Medicine.

[11] Jian Q, Xiaojun D, Zhoutong L, Hanqing Z, Jie Q, Hua C (2015). Clinical study on preventive administration of Danyu Gukang Pill to prevent femoral head necrosis after femoral neck fracture. Henan Traditional Chinese Medicine.

[12] Ning K, Zhao X, Poetsch A, Chen WH, Yang J (2017). Computational molecular networks and network pharmacology. Biomed Res Int.

[13] Ye H, Wei J, Tang K, Feuers R, Hong H (2016). Drug Repositioning through network pharmacology. Curr Top Med Chem.

[14] Zhang W, Bai Y, Wang Y, Xiao W (2016). Polypharmacology in drug discovery: a review from systems pharmacology perspective. Curr Pharm Des.

[15] Kanehisa M, Goto S (2000). KEGG: kyoto encyclopedia of genes and genomes. Nucleic Acids Res.

[16] Kanehisa M (2019). Toward understanding the origin and evolution of cellular organisms. Protein Sci.

[17] Kanehisa M, Furumichi M, Sato Y, Ishiguro-Watanabe M, Tanabe M (2021). KEGG: integrating viruses and cellular organisms. Nucleic Acids Res.

[18] Moya-Angeler J, Gianakos AL, Villa JC, Ni A, Lane JM (2015). Current concepts on osteonecrosis of the femoral head. World journal of orthopedics.

[19] Baig SA, Baig MN (2018). Osteonecrosis of the femoral head: etiology, investigations, and management. Cureus.

[20] Liu ZH, Sun XB. Network pharmacology: new opportunity for the modernization of traditional Chinese medicine. Yao xue xue bao = Acta pharmaceutica Sinica. 2012;47(6):696–703. 22919715

[21] Yu T, Zhang Z, Xie L, Ke X, Liu Y (2016). The influence of traditional Chinese medicine constitutions on the potential repair capacity after osteonecrosis of the femoral head. Complement Ther Med.

[22] Zhang Q, Yang F, Chen Y, Wang H, Chen D, He W (2018). Chinese herbal medicine formulas as adjuvant therapy for osteonecrosis of the femoral head: a systematic review and meta-analysis of randomized controlled trials. Medicine.

[23] Boezio B, Audouze K, Ducrot P, Taboureau O. Network-based approaches in pharmacology. Mol Inform. 2017;36(10). 10.1002/minf.201700048 28692140

[24] Ru J, Li P, Wang J, Zhou W, Li B, Huang C (2014). TCMSP: a database of systems pharmacology for drug discovery from herbal medicines. Journal of cheminformatics.

[25] Wong SK, Chin KY, Ima-Nirwana S (2020). Quercetin as an agent for protecting the bone: a review of the current evidence. Int J Mol Sci.

[26] Pang XG, Cong Y, Bao NR, Li YG, Zhao JN (2018). Quercetin stimulates bone marrow mesenchymal stem cell differentiation through an estrogen receptor-mediated pathway. Biomed Res Int.

[27] Zhou Y, Wu Y, Jiang X, Zhang X, Xia L, Lin K (2015). The effect of quercetin on the osteogenesic differentiation and angiogenic factor expression of bone marrow-derived mesenchymal stem cells. PLoS ONE.

[28] Hong JM, Kim TH, Chae SC, Koo KH, Lee YJ, Park EK (2007). Association study of hypoxia inducible factor 1alpha (HIF1alpha) with osteonecrosis of femoral head in a Korean population. Osteoarthritis Cartilage.

[29] Ma W, Xin K, Chen K, Tang H, Chen H, Zhi L (2018). Relationship of common variants in VEGFA gene with osteonecrosis of the femoral head: a Han Chinese population based association study. Sci Rep.

[30] Yu J, Liang F, Huang H, Pirttiniemi P, Yu D (2018). Effects of loading on chondrocyte hypoxia, HIF-1alpha and VEGF in the mandibular condylar cartilage of young rats. Orthod Craniofac Res.

[31] Xu J, Sun Y, Wu T, Liu Y, Shi L, Zhang J (2018). Enhancement of bone regeneration with the accordion technique via HIF-1alpha/VEGF activation in a rat distraction osteogenesis model. J Tissue Eng Regen Med.

[32] Yan Z, Hang D, Guo C, Chen Z (2009). Fate of mesenchymal stem cells transplanted to osteonecrosis of femoral head. J Orthop Res.

[33] Potier E, Ferreira E, Andriamanalijaona R, Pujol JP, Oudina K, Logeart-Avramoglou D (2007). Hypoxia affects mesenchymal stromal cell osteogenic differentiation and angiogenic factor expression. Bone.

[34] Riddle RC, Khatri R, Schipani E, Clemens TL (2009). Role of hypoxia-inducible factor-1alpha in angiogenic-osteogenic coupling. J Mol Med (Berl).

[35] Zou D, Han W, You S, Ye D, Wang L, Wang S (2011). In vitro study of enhanced osteogenesis induced by HIF-1α-transduced bone marrow stem cells. Cell Prolif.

